# Losing the genetic twin: donor grief after unsuccessful unrelated stem cell transplantation

**DOI:** 10.1186/1472-6963-9-2

**Published:** 2009-01-07

**Authors:** Martina Wanner, Sandra Bochert, Iris M Schreyer, Gabi Rall, Claudia Rutt, Alexander H Schmidt

**Affiliations:** 1 DKMS German Bone Marrow Donor Center, Kressbach 1, 72072 Tuebingen, Germany

## Abstract

**Background:**

Stem cell transplantations from related or unrelated donors are used to cure leukaemia and other blood diseases. When a patient dies after an unsuccessful transplantation, interested unrelated donors are informed about the failure by their donor centre. Studies focussing on failed related donations show that donors undergo an intense grieving process. As there are only two investigations about reactions from unrelated donors, knowledge about their reactions is less comprehensive.

**Methods:**

We conducted a prospective study of reactions of unrelated donors to the information of failed transplantations, subject to various communication methods (letter, phone). Questionnaires were sent to 395 unrelated donors who received the news of their recipients' deaths between November 2005 and August 2006. In addition, twelve in-depth interviews with selected donors were carried out.

**Results:**

Unrelated donors were emotionally affected by the recipients' deaths, and it is appropriate to speak about a "Donor Grief" phenomenon, as the results of 325 returned questionnaires (return rate 82.3%) and in-depth interviews show. Donors demonstrated a range of feelings such as sadness, disappointment, grief, and helplessness. These feelings were often unexpectedly intense given the fact that the recipient was a stranger. Although the news caused grief, donors underlined that they nevertheless wanted to be informed. They preferred knowledge of the failure to uncertainty. The method of providing the information is only of secondary importance. Most donors favoured the way of communication they had experienced.

**Conclusion:**

This result indicates that both phone and letter communication can be justified. However, phone communication seems to be superior with respect to aspects of sensitivity. In spite of transplantation failure and the associated negative feelings, most donors were happy to have donated and would be willing to do so again. Our results underline the special responsibility of donor centres for informing and supporting unrelated volunteer donors in case their recipients have died.

## Background

Unrelated haematopoietic stem cell transplantation is an increasingly used treatment for leukaemia and other diseases of the blood [1,2]. In stem cell transplantation, a close human leukocyte antigen (HLA) match between donor and recipient correlates positively with transplantation outcome [3,4]. Donor searches generally start within the recipients' families [5]. However, most recipients in need of stem cell transplantation do not find a matching family donor. They depend on a match with an unrelated volunteer who is willing to donate stem cells to a stranger. Due to the considerable variability of the HLA system [6], large files of potential stem cell donors were established. Today, these files comprise more than 12,000,000 potential stem cell donors worldwide [7]. Donor centres are responsible for recruiting, administering, and guiding potential donors as well as facilitating stem cell donations.

Stem cell donors undergo one of two methods, peripheral blood stem cell donation or bone marrow donation. Both methods may lead to discomfort and are associated with small but existing risks [8].

Stem cell transplantations are not always successful. Some recipients die as a result of their diseases, some from transplantation-related complications. The success rate of stem cell transplantations depends on factors such as the recipient's disease, disease status, comorbidity, age, and the degree of HLA match between recipient and donor. In addition, donor age, gender, as well as cytomegalovirus and Epstein-Barr virus serology are of potential relevance for transplantation success. In view of the efforts donors undergo, donor centres should try to cater to donors' wishes to be updated about their recipients' states of health. Thus, donor centres should develop policies regarding communication of recipients' deaths to their donors.

Studies show that most donors associate positive feelings with the donation. They are happy and proud for having donated and are willing to donate again [9-15]. Unrelated donors demonstrate even more positive feelings than related donors [16]. Helping behaviour and altruism are central motives. Helping others seems to be fulfilling and self-gratifying for the help-giver [13,14]. However, possible negative outcomes of stem cell transplantations like recipients' deaths cause grief to donors [17,18].

Grief can have a strong influence on the well-being of a person and affect physical sensations as well as perceptions, and cause various patterns of behaviour. Feelings such as sadness, anger, self-reproach, anxiety, loneliness, helplessness, shock, yearning, and guilt are evoked [19-23]. The death of a close person in particular is a critical event in life which demands a certain readjustment. "Mourning work" is necessary to accept the loss and to return to normality [24,25]. In the description of readjustment, several theories have been developed [26,27].

In the case of an unsuccessful related stem cell donation, many donors grieve, feel responsible, and blame themselves for the deaths of their relatives [15,28-34].

Unrelated stem cell donors, however, do not have a personal relationship to their recipients, and, therefore, do not have the same potential for loss as related donors. Donor and recipient are strangers. They are allowed to exchange letters anonymously via the donor and transplant centres. Two years after donation, anonymity between donors and recipients can be lifted if both sides agree and no legal restrictions of the recipient country require ongoing anonymity.

Only two studies investigated the effect of deaths of recipients on unrelated donors. Feelings of responsibility, guilt, shock, disappointment, and sadness occur among unrelated donors and are surprisingly intense, as a study of the National Marrow Donor Program (NMDP) of the U.S.A. points out. This study was based on a secondary data analysis of follow-up questionnaires after stem cell transplantations and on additional in-depth interviews [17]. Therefore, bad news must be broken carefully. A recent retrospective study of the Welsh Bone Marrow Donor Registry (WBMDR) concludes that most donors prefer to be informed by phone [18] as the loss of the recipient affects them and they need guidance. The findings of the two studies emphasise the responsibility of donor centres to sensitively support their donors in this critical situation. However, both studies show limitations through their respective study designs. It is the aim of our study to gain more comprehensive knowledge of experiences of donors regarding the donation and the deaths of recipients in case of an unsuccessful transplantation through a large donor panel, a prospective study design, and the combination of quantitative and qualitative elements. The results shall lead to the most appropriate method of informing donors about their recipients' deaths and to the best way of supporting them in this situation.

One might argue that donor centres could avoid this critical situation by not informing donors about their recipients' deaths. However, there is general agreement that donor centres should inform their interested donors on the health states of their respective recipients. This also applies to the information about recipients' deaths [35,36].

## Methods

### Subjects and procedure

Subjects for the prospective study were 395 donors who donated stem cells between 2003 and 2005 and wanted to be informed about the states of health of their recipients. They received news of their recipients' deaths between November 2005 and August 2006. Donors who already had non-anonymous contact with their recipients were not included in the study.

All donors were randomly assigned to one of three groups. Corresponding to the DKMS standard procedure, donors of Group A were informed by letter. The letter consists of the following elements: preparation for bad news, information about the death of the recipient, cause of death, recollection of the fact that there is generally a considerable risk of an unsuccessful transplantation, sincere thanks to the donor. Regarding the cause of death, we differentiate between four items: "pneumonia", "infection", "relapse" and "cause unknown". Group B donors receive a very similar letter, apart from an explicit invitation to make a return telephone call if the donor wishes to talk about the death of the recipient to a DKMS employee. All donors who made a return call talked to the same person (MW) in order to standardise the procedure. Donors of Group C were informed by telephone directly. The phone conversation consisted of the same elements as the letters. The duration of the conversations was highly variable due to different donor reactions. It ranged from a few to approximately 30 minutes. Again, all phone calls were made by the same person (MW).

Approximately four weeks after having received the information all donors were asked to provide feedback via a questionnaire. A second, identical questionnaire was sent as a reminder to those donors who did not answer within four weeks. The questionnaire includes a statement of informed consent to be signed by responding donors. To validate the questionnaire, a pilot study with 107 donors was carried out (see paragraph *Questionnaire*). A slightly adapted questionnaire was then sent to the remaining 288 donors. In this article, we analyze the panel of all 325 respondents (112 from Group A, 110 from Group B, and 103 from Group C) who returned the questionnaire (total return rate 82.3%). Donor background characteristics were collected to determine whether they influence donor reactions regarding recipients' deaths.

Additionally, we conducted one-hour in-depth face-to-face interviews with 12 donors. Only donors living in Baden-Wuerttemberg were considered for face-to-face interviews in order to reduce travel costs. Among the 46 respondents from that state, donors with uncommon or exceptional answers were preferably chosen. The interviewees also signed an informed consent statement.

The study was approved by the Institutional Review Board of the State Medical Chamber of Baden-Wuerttemberg.

### Questionnaire

A 9-item questionnaire (see Additional file 1) was used. 4 questions (Questions 1, 3–5) address the process of being informed about the recipient's death, 3 questions (Questions 6–8) are related to donor emotions and coping strategies. Furthermore, we asked how well donors felt informed prior to the donation about the possibility that their recipients could die (Question 2) and if they would donate again for another recipient (Question 9). 6 questions allow for comments.

After having defined the goals of the study, we developed the questionnaire in three steps: First, we checked if a validated questionnaire existed that we could use. This was not the case, mainly due to the specific nature of the study, particularly its focus on the optimal way to inform unrelated stem cell donors about their recipients' deaths. Second, we internally discussed feedback of donors whose recipients had died. Goal of these discussions was to extract typical donor reactions to transplantation failure and to the method of communication. These patterns laid the main foundation of the questionnaire. It has to be noted that, prior to our study, we did not systematically ask for donor feedback in this area. Instead, donors provided feedback as part of their usual communication with donor centre staff. Steps 1 and 2 partially overlapped in time. Third, we carried out a pilot to check the questionnaire and the data collection process. Specific issues addressed in the pilot phase were: Is the return rate satisfying? Are there indications that certain questions are unclear or dispensable? Are there any issues raised in donor comments that should be included into the questionnaire?

The pilot return-rate of 86.9% (93/107) was satisfying. Changes in the questionnaire were only made to Question 8: Since only 4 donors who agreed to the statement "I often think about the family of the recipient" did not agree to "I often think about the recipient", these items were combined to "I often think about the recipient or the family of the recipient" in order to reduce redundancy. Furthermore, three items were added (see Additional file 1) as they were given in donor comments.

### Statistical analysis

To more clearly determine the tendency of the responses, all questions with 4- or 6-point scales were dichotomized (1/2 versus 3/4 or 1–3 versus 4–6, respectively).

For each question we tested for significant differences between answers given by donors who were contacted by letter or phone, respectively. To account for multiple testing, results with *p *< 0.005 were considered to be significant.

Furthermore, we searched for correlations between specific answers. For this purpose, we developed 5 hypotheses. These hypotheses were: (a) Donors who feel they were poorly informed prior to the donation also view DKMS' communication process with regards to recipients' deaths more negatively than other donors; (b) Donors who feel well informed prior to the donation are less affected emotionally than other donors; (c) Donors who are not affected emotionally feel more often than other donors that they were poorly informed about the cause of death; (d) Donors who are not affected emotionally prefer to be informed by letter more often than other donors; (e) Donors who are affected emotionally are less often willing to donate again than other donors. Results were considered to be significant for *p *< 0.05.

Additional data were collected in order to determine correlations between answers to the questionnaire and donor background characteristics or other potential influencing factors (see Table 1). The impact of the various factors was analyzed by applying logistic regression analysis. For Question 1, Answers 2 (bad) and 3 (I do not know) were summarised as "sceptical" in the logistic regression analysis. Answers 2 (no) and 3 (I do not know) of Question 9 were treated equivalently. Regression analysis was carried out with SPSS 14.0. Due to multiple testing, results with *p *< 0.0005 were considered to be significant.

**Table 1:** Donor background characteristics

**Background characteristics**		**Number**	**Percentage**
Donor gender	Female	97	29.8
	Male	228	70.2
Donor age	< 30	99	30.5
	≥ 30	226	69.5
Population density	< 100	55	16.9
	≥ 100	270	83.1
Regions	South	156	48.0
	North	169	52.0
Recruitment	Recruitment related to a patient	237	72.9
	Recruitment not related to a patient	88	27.1
Recipient age	< 15	90	27.7
	≥ 15	232	71.4
	Not available	3	0.9
Anonymous contact	Yes	49	15.1
	No	276	84.9
Communication	Letter	222	68.3
	Phone	103	31.7

χ^2 ^tests were used as significance tests. If statistical pre-conditions for χ^2 ^tests were not fulfilled Fisher's exact test was applied.

### In-depth interviews

We conducted 12 face-to-face interviews using a semi-standardised guideline. Donors were invited to describe the experiences they made during the process, from recruitment through donation to the post-donation follow-up. Experiences with the news of the recipient's death were emphasised.

We based the interview analysis on the principles of the Grounded Theory approach [37]. Interview transcripts were analysed line by line. We carried out several coding steps in order to elaborate central donor statements.

## Results

### Quantitative analysis of donor responses

Most donors consider being informed about deaths of recipients as positive (300/321, 93.5%; Question 1). Only 2.2% (7/321) of donors consider being informed negative, and 4.4% (14/321) do not know. This result is not surprising, as only donors who asked to be informed about their recipients' health states were included in the study.

258 of 318 donors (81.1%) feel well informed about the possibility that the recipient could die after the treatment (Question 2). This result reflects the efforts undergone by DKMS employees to inform potential donors about transplantation risks. However, a considerable number of donors does not feel optimally informed. Possible reasons are that information provided by DKMS was not sufficient or that donors generally tend to underestimate the severity of patients' health states and/or the risks of transplantation. Statements from donors in the interviews suggest that both reasons might play a role (see *In-depth interviews *paragraph).

Most donors were content with the way they received information about the death of their recipient (Question 3). 163 of 215 donors who were informed by letter preferred this method (Groups A and B, 75.8%) while 76 of 101 donors who were informed by telephone (Group C) preferred a phone call (75.2%). Preferences for phone calls were significantly higher in Group C (χ^2 ^test, *p *< 0.001). On the other hand, as many donors from Groups A/B as from Group C preferred the way of communication they had experienced (χ^2 ^test, *p *= 0.76). Therefore, it is not possible to identify a general superiority of one method of communication. This is also supported by the fact that only 10 Group B donors and 5 Group A donors contacted DKMS by phone.

Donors are also satisfied with the communication process itself (Question 4). They state that it was not inadequate (239/252, 94.8%), it was sensitive (254/281, 90.4%), helpful (217/270, 80.4%), and informative (202/274, 73.7%). Compared to Group A and B donors, fewer Group C donors regard the way of communication as inadequate (Fisher's exact test, *p *= 0.01) while significantly more Group C donors classify it as sensitive (χ^2 ^test, *p *= 0.003).

The cause of the recipient's death ("pneumonia", "infection", or "relapse") was communicated to 150 donors of our sample. Answers to Question 5 show that donors do not perceive the information to be dispensable (95/96, 99.0%) and find it understandable (102/111, 91.9%) and sufficient (84/104, 80.8%). Although most donors find it helpful to be informed about recipients' deaths (see Question 1), 42.7% of donors (41/96) find the information depressing. Comparing the three groups, we find that a larger number of Group C donors find the information understandable (Fisher's exact test, *p *= 0.005) and sufficient (χ^2 ^test, *p *= 0.02). Our strict significance criteria, however, are not fulfilled for these two items. For the items "dispensable" and "depressing", no relevant differences can be found.

Although the recipient was a stranger, 299 of 313 donors (95.5%) are emotionally affected by her or his death (Question 6). Donors who are affected report a wide spectrum of feelings (Question 7): They are sad (280/292, 95.9%) and disappointed (253/290, 87.2%). In addition, they feel grief (208/284, 73.2%) and helplessness (140/270, 51.9%). Only few donors are shocked (78/267, 29.2%) or show a lack of comprehension (44/255, 17.3%). These results demonstrate the intensity of donors' feelings regarding the deaths of their recipients.

With regards to specific coping behaviours (Question 8), 88.0% of donors (66/75) of the pilot study and 88.6% of donors (186/210) of the main study disagree with the statement "I do not think about it". It shows that donors do think about the recipients and their families. In the pilot study, 68.7% of donors (57/83) agree with the statement "I often think about the recipient" and 58.3% (49/84) with "I often think about the family of the recipient". In the main study, 64.3% of donors (135/210) agree with "I often think about the recipient or the family of the recipient". Donors also tend to think more often about their own families: 77.6% of donors of the pilot study (59/76) agree with "I think about my own family more often". 51.9% of donors of the main study (108/208) agree with a very similar statement. The main study shows that most donors (140/214, 65.4%) talk to relatives and friends as coping strategy. However, donors normally do not approach other persons. Most of them do not want to contact DKMS (188/206, 91.3%) or other donors (195/205, 95.1%). Donors from Groups A and B state more often that they want to contact DKMS (χ^2 ^test, *p *= 0.01). A very remarkable finding is that most donors agree with the statement "Nevertheless, I feel happy for having donated" (pilot study: 86/92, 93.5%; main study: 224/229, 97.8%). The fact that many donors use the abovementioned coping strategies underlines the fact that they are emotionally affected.

As most donors feel positive about having donated despite the negative transplantation outcome, it is not surprising that most donors are willing to donate again (291/322, 90.4%; Question 9). 25 donors (7.8%) are unsure if they would donate again, 6 donors (1.9%) would not donate again.

Whenever we did not point out any differences between Groups A/B and C in this paragraph, the corresponding *p *values are greater than 0.05.

### Correlations between answers

Donors who feel they were poorly informed prior to donation (Question 2) consider the communication about the recipients' deaths (Question 4) significantly less often as helpful (χ^2 ^test, *p *< 0.01) and informative (χ^2 ^test, *p *= 0.01) compared to donors who feel well prepared. For the item "sensitive", the result is close to significance (Fisher's exact test, *p *= 0.06). This result suggests that unsatisfactory communication before the donation can lead to negative donor experiences even at a much later date. Alternatively, the respective donors could generally have a critical attitude with regards to DKMS' procedures.

We hypothesized that donors who felt well informed prior to donation (Question 2) showed less emotional affectedness (Question 6) than other donors. This would be plausible since well-informed donors should know that a negative transplantation outcome might occur with considerable probability. Indeed, all 14 donors with no emotional affectedness belong to the group that felt well informed while all 60 donors who felt poorly informed prior to transplantation are emotionally affected. However, differences are statistically not significant (Fisher's exact test, *p *= 0.08).

We also hypothesized that more donors without emotional involvement (Question 6) preferred to be informed by letter (Question 3) than other donors. Data seem to support this. The results are, however, not significant (χ^2 ^test, *p *= 0.14). If we only consider donors who state to be emotionally affected, we find that donors who do not feel grief (Question 7) prefer to be informed by letter significantly more often than other donors (χ^2 ^test, *p *< 0.01). There is no such correlation for the other items of Question 7 (sadness, helplessness, disappointment, shock, and lack of comprehension). Therefore, it can finally not be concluded if it is especially appropriate for donors with a tendency to show little emotional involvement to provide information about the death of a recipient by letter.

For the other tested hypotheses (see *Methods *section), no significant correlations could be identified (Fisher's exact test, *p *> 0.4).

### Association of donor responses with background characteristics and type of contact

Table 1 summarizes the frequencies of various background characteristics of responding donors. Groups A, B, and C differ significantly only with regards to their regional composition (χ^2 ^test, *p *= 0.02).

Table 2 shows all correlations between donor answers and background characteristics with *p *< 0.05 as provided by the logistic regression analysis. The only significant finding is the higher preference for being informed via phone by donors who were informed this way. Some findings with *p *< 0.05, although not adhering to our strict significance criteria of the logistic regression analysis, may nevertheless indicate relevant correlations: Items #3 and #5 are also significant (sensitive communication, *p *= 0.003) or close to significant (sufficient information, *p *= 0.02) in the respective univariate tests (see paragraph *Quantitative analysis of donor responses*). The first of these correlations could be explained by the more personal approach given by phone communication, the second by the opportunity to directly ask questions. Items #4, #7, #8, and #12 may refer to some gender-specific appraisals. The correlation suggested by Item #13 also seems plausible as the respective donors might already have established a closer relationship with their recipients. Furthermore, the wish to have anonymous contact may indicate a special interest of donors in their recipients' well-being.

**Table 2:** Results of logistic regression analysis.

**#**	**Question**	**Statement/item**	**Factor**	***p *value**	**Odds ratio**	**99% confidence interval**
1	2	Feeling well prepared prior to donation	Donor age (< 30)	0.03	2.11	0.85–5.27
2	3	Preferring phone communication	Contact (phone)	< 0.0001*	9.16	4.38–19.16
3	4	Assessing communication as sensitive	Contact (phone)	0.02	6.04	0.86–42.33
4	4	Assessing communication as sensitive	Donor gender (female)	0.02	4.41	0.81–24.04
5	5	Assessing information as sufficient	Contact (phone)	0.01	5.30	0.97–29.00
6	7	Feeling grief	Population density (< 100)	0.05	2.40	0.77–7.48
7	7	Feeling helplessness	Donor gender (female)	0.04	1.76	0.85–3.62
8	8 (P)	Often thinking about the family of the recipient	Donor gender (female)	0.04	4.83	0.65–35.93
9	8 (P)	Often thinking about the family of the recipient	Circumstance of recruitment (not related to patient)	0.04	3.34	0.74–14.99
10	8 (P)	More often thinking about own family	Donor age (≥ 30)	0.03	4.81	0.78–29.54
11	8	Not thinking about it	Circumstance of recruitment (not related to patient)	0.01	3.52	1.00–12.36
12	8	Often thinking about the recipient/the family of the recipient	Donor gender (female)	0.002	3.03	1.21–7.59
13	8	Often thinking about the recipient/the family of the recipient	Anonymous contact with recipient (yes)	0.04	2.67	0.80–8.91
14	8	More often thinking about own family since reception of the news	Contact (letter)	0.05	1.85	0.82–4.16
15	8	Wanting to contact DKMS	Contact (letter)	0.03	9.68	0.64–146.31

All combinations of answers and factors with p values < 0.05 are displayed. Due to multiple testing, only p values < 0.0005 are considered as significant and marked with an asterisk in the table. Examples: Donors who have been contacted by phone significantly more often prefer phone communication than other donors (#2). Female donors more often state to often think about the recipient or his family than male donors but the difference is not regarded as significant (#12). P = Pilot.

### Qualitative data – Questionnaire

184 donors added 447 comments to the questionnaires (56.6% of all responding donors). However, comments often do not reveal any new aspects. Instead, donors emphasize a specific topic, e.g., they underline their emotional involvement. Most comments relate to the preparation for the donation (Question 2, 125 comments). Donors provide positive feedback when they feel they were well informed about the possibility that the recipient could die (29 comments) and criticise when they do not feel they were well informed (23 comments). Donors also often explain their coping strategies (Question 8, 102 comments). When receiving the news of recipients' deaths, donors are supported by family members and friends (39 comments). The possibility to contact DKMS is also positively acknowledged (35 comments) – even if the opportunity is not often used. 14 donors note that anonymity between donor and recipient helps them to deal with the news. To avoid inducing responsibility and guilt, we did not ask donors if they blamed themselves for the recipients' deaths. Nevertheless, nine donors state to feel guilty. 11 of the 25 donors who note that they are unsure whether to donate again and all six donors who withdrew their willingness to donate (Question 9) provide additional comments to explain their motives. Explanations include: burden of the donation, health risks, personal situation, and disappointment.

### Qualitative data – In-depth interviews

In the semi-structured face-to-face interviews, donors (Table 3) described their experiences with the donation and the feelings they had when they got the news of the recipients' deaths with their own words. To be the suitable donor for a patient was a very meaningful event for all 12 donors. One interviewee explained: "To rescue life is something very special. You do not often get the chance to save a life". (#10) Donors are happy to be able to help someone. One woman characterises her feelings: „I was as pleased as Punch to be able to help someone. That was unbelievable!” (#9) For one donor the possibility to help was a present even for himself. His stem cell donation took place a few days before Christmas and was the greatest gift for him (#6). One of the donors even compared his donation with the birth of a child: "Now I am able to understand feelings a woman must have when she bears a child. Childbirth must be a very painful event but when the child is born, everything is forgotten. Perhaps it is a poor comparison because childbirth is much more miraculous, but I had similar feelings". (#12)

**Table 3:** Selected background characteristics of interviewees

**Donor #**	**Donor gender**	**Donor age**	**Recipient age**	**Anonymous contact**	**Communication**
1	female	46	49	No	Phone
2	male	32	11	No	Letter
3	male	28	64	No	Letter
4	male	53	64	No	Letter
5	male	38	42	No	Letter
6	female	32	5	No	Letter
7	male	29	57	No	Letter
8	male	27	36	No	Phone
9	female	34	40	No	Letter
10	male	44	13	Yes	Phone
11	male	43	14	No	Letter
12	male	41	62/54 (two donations)	No	Letter

All combinations of answers and factors with p values < 0.05 are displayed. Due to multiple testing, only p values < 0.0005 are considered as significant and marked with an asterisk in the table. Examples: Donors who have been contacted by phone significantly more often prefer phone communication than other donors (#2). Female donors more often state to often think about the recipient or his family than male donors but the difference is not regarded as significant (#12). P = Pilot.

All interviewees wanted to know as much as possible about the person who received their cells. One donor reported on the preparation in the hospital: "The physician asked me how much I wanted to know and my answer was: As much as you are allowed to tell me". (#2) Donors showed a special personal interest in the recipient; they were concerned about him: "I tried to imagine what kind of person he was. I asked myself for how long he might have been ill and whether he had a family who hoped for his recovery". (#9) The interviewees often thought about the recipients. 11 of the 12 donors said that there might be more to it than just the matching of HLA characteristics. One female donor explained: "They said that it was an American woman who was even close to my age. (...) This was interesting for me. I thought about what she would look like, and what kind of person she would be? Perhaps we had similarities?" (#1) Another donor reported that his brother-in-law who also donated stem cells had some similarities with his Canadian recipient. They look similar and have the same taste (#2). Donors think that there is a special relationship and that they share similarities with the recipient: "Well, I felt like I was closely connected to him". (#9)

Given this background, it is not astonishing that donors were affected by recipients' deaths: „The news made me very sad. I really hoped that my stem cells would help!” (#4) Another donor was depressed: "It was my deepest hope to help the boy. (...) I wished he would win the battle. When I got the news of his death I was very, very sad. My wife even cried". (#11) Ten donors expressed that they had feelings such as grief. But emotional involvement implies that there is more to it than the fact that a person has died. One female donor said: "Our relationship has been broken, although there never was a ‚real relationship’ between us. But I knew that there was someone who got something from me and this should have saved his life! But it did not work. I asked myself why it did not work and I found no answer to this question". (#9) Only one donor was not affected by the death of the recipient. He explained this attitude with his occupation. As a healthcare professional he was faced with severe suffering every day and was used to dealing with death and dying (#7).

Four interviewees said they did not underestimate the severity of their recipients' health states and the risks of transplantation (#1, #6, #7 and #8), four interviewees pointed out their own unrealistic view (#4, #9, #10, #11), three interviewees criticised that the information provided by DKMS was not sufficient (#2, #3, #12) and Interviewee #5 did not comment on this issue.

All interviewees explained that the attempt to save the life of the recipient is worthwhile the effort. They underline that they have done their part and tried to help. Everyone – except for one woman who is currently starting a family – would donate again.

When asked which procedure of being informed they would have preferred, the donors always chose the method they were used to.

## Discussion

Our study investigates emotional affectedness and grief reactions in the setting of unrelated stem cell donation and shows that most unrelated donors were emotionally affected by their recipients' deaths. Feelings were often unexpectedly intense given the fact that the recipients were strangers. The use of specific coping behaviours and the fact that several donors stated to feel guilty underline this involvement. Our findings show that it is appropriate to speak about a "Donor Grief" phenomenon. Furthermore, they confirm the results of the only two existing studies that focus on reactions of unrelated donors on recipients' deaths [17,18].

Considering the question of why unrelated donors are emotionally highly affected, we were able to develop two hypotheses based on the findings from the in-depth interviews:

First, grief reactions may result from the supposed exceptional relationship between donor and recipient. 11 of the 12 interviewed donors wondered if they shared characteristics beyond HLA with their recipients and were their "genetic twins". In this perceived special relationship lies a potential for emotional affectedness and grief reactions [22]. Further research is necessary to determine this issue more clearly: How do unrelated stem cell donors characterise the relationship with and the perceived similarities to their recipients?

Second, having the opportunity to help as stem cell donors was a very special event for interviewed donors. They got the rare chance to save another person's life. As confirmed by other studies [10-12], donors showed a high level of altruism and were deeply disappointed when the transplantation failed. The lost opportunity to perform an important act even for a stranger caused grief [13,14].

Donors' hopes regarding the positive outcomes of transplantation may be unrealistic. It is, therefore, important that donor centre professionals give a realistic appraisal during donation preparation and inform openly about the possibility of failure. Donors who feel poorly informed prior to donation significantly less often consider the communication regarding the recipient's death to be helpful and informative.

Our study donors expressed their desire for information and criticised if they felt insufficiently updated. This finding confirms an earlier study showing that donors preferred the knowledge of the failure to uncertainty even if this knowledge caused grief [17]. It is, therefore, no option for donor centres not to inform donors about their recipients' deaths in order to avoid feelings of grief.

Each of the two possible communication methods – in writing or by phone – has its advantages. Receiving a letter enables donors to first reflect on the news. They do not have to react immediately. Furthermore, the letter can be shown to friends and family members. Providing the information by phone offers a more personal approach. Donors can get immediate support from donor centre professionals and can easily ask for more detailed information. Our findings suggest that there are no general donor preferences for either of the two methods. Most DKMS donors favoured the method they were used to. This fact may result from donors' own positive experiences. We conclude that both methods are accepted by donors when carried out carefully. This finding is contradictory to the results of the WBMDR study that identifies a clear donor preference for a direct personal approach (phone calls or face-to-face contact [18]). It is unclear to which extent such differences might be induced by cultural differences between the two populations from Wales and Germany, respectively.

Our study also shows small differences in donor experiences: Donors who were informed by phone more often consider the method adequate and significantly more often sensitive. They also more often consider the information understandable and sufficient. Donor centres should be aware of these differences and offer possibilities to ask questions and obtain support. Every donor centre has to decide whether to use considerable additional resources to gain the small benefits of phone calls. The role of donor centre professionals in this respect should also not be over-estimated. Our study shows that donors develop their own coping strategies, e.g., getting help from family members and friends. The relevance of these personal resources is also emphasised in the literature [38]. Donors should be encouraged to refer to them.

The fact that most donors have positive donation experiences and would be willing to donate again has often been pointed out in the literature [9-16]. No studies so far have analysed the effect of transplantation success on donors' appraisal of the donation in the setting of unrelated donation. One might hypothesise that a negative transplantation outcome also leads to a more negative assessment of the donation experience. Our findings, however, demonstrate that most donors were happy to have donated and would be willing to donate again although their recipient has died. Stem cell donation seems to have a fundamental importance for donors. The return rate of the questionnaire of 82.3% also documents the interest in stem cell donation and the study focus.

## Conclusion

Although our questionnaire has not been used in studies before, we conclude that our study with the large donor panel, the prospective design, the combination of quantitative and qualitative methods, and the high return rate can be seen as an important step to gain a better understanding of the grieving process of unrelated stem cell donors. Prior to donation, donors should be given a realistic appraisal of transplantation prospects. In case of recipients' deaths donors show unexpectedly intense feelings, and donor centres have to develop appropriate ways to face the "Donor Grief" phenomenon. Although phone communication seems to be superior to written communication with regards to sensitivity, both methods can be justified. However, it is important to give donors the chance to ask questions and call for support. Our findings may guide donor centres to optimize their processes of informing donors about recipients' deaths and of supporting them.

## Competing interests

The authors declare that they have no competing interests.

## Authors' contributions

GR and CR initiated the study. All authors made substantial contributions to the study design. MW informed donors by phone, IMS informed donors by letter. MW interviewed selected donors. MW and SB collected data. MW and AHS conducted analyses and wrote the manuscript. All authors read and approved the final manuscript.

## Pre-publication history

The pre-publication history for this paper can be accessed here:



## Supplementary Material

Additional file 1 **Questionnaire**. Click here for file
