# Supplementary material for: Losing the genetic twin: donor grief after unsuccessful unrelated stem cell transplantation
Source: BMC Health Serv Res. 2009 Jan 7;9:2. doi: 10.1186/1472-6963-9-2 (PMC2630924; doi:10.1186/1472-6963-9-2)
Supplement: Additional file 1 — Questionnaire. [file 1472-6963-9-2-S1.doc]

**Questionnaire**

Please mark for the following questions the answers that apply best to you. There are no “right” or “wrong” answers. Please skip questions you do not want to answer.

1. How do you basically assess it to be informed on the patient’s death?

O good

O bad

O I do not know

1. If you remember the time prior to your stem cell donation: How well or badly did DKMS’ information prepare you for the possibility that the patient could die? Please choose on a scale from 1 to 6.

|  | 1 | 2 | 3 | 4 | 5 | 6 |  |
| --- | --- | --- | --- | --- | --- | --- | --- |
| Excellent |  |  |  |  |  |  | Unsatisfactory |

Comment: ___________________________________________________________

____________________________________________________________________

____________________________________________________________________

1. Which procedure do you think is adequate?

O to be informed by a letter

O to be informed by a phone call

1. How did you perceive the way DKMS communicated the message to you?

|  | not | little | quite | very |
| --- | --- | --- | --- | --- |
| helpful |  |  |  |  |
| inadequate |  |  |  |  |
| informative |  |  |  |  |
| sensitive |  |  |  |  |

Comment: ___________________________________________________________

____________________________________________________________________

____________________________________________________________________

1. Unfortunately, we do not always know the cause of death of a patient. If you have **not been informed** on the cause of death, **please go directly to Question 6**. However, if you have been informed on the cause of death, was the information

|  | not | little | quite | very |
| --- | --- | --- | --- | --- |
| understandable |  |  |  |  |
| depressing |  |  |  |  |
| sufficient |  |  |  |  |
| dispensable |  |  |  |  |

Comment: ___________________________________________________________

____________________________________________________________________

____________________________________________________________________

1. Did the patient’s death affect you emotionally? **If not, please go directly to Question 8.**

O Yes

O No

- 1. **If yes**, please mark which feelings you had when you were informed about the patient’s death.

When I received the message, I felt…

|  | disagree strongly | disagree | agree | agree strongly |
| --- | --- | --- | --- | --- |
| grief |  |  |  |  |
| sadness |  |  |  |  |
| helplessness |  |  |  |  |
| disappointment |  |  |  |  |
| shock |  |  |  |  |
| lack of comprehension |  |  |  |  |

Comment: ___________________________________________________________

____________________________________________________________________

____________________________________________________________________

**PILOT**

1. Please find below some ways to deal with such a message. Please mark how the statements apply to you.

|  | disagree strongly | disagree | agree | agree strongly |
| --- | --- | --- | --- | --- |
| I do not think about it |  |  |  |  |
| I often think about the recipient |  |  |  |  |
| I often think about the family of the recipient |  |  |  |  |
| I think about my own family more often |  |  |  |  |
| Nevertheless, I feel happy for having donated |  |  |  |  |

The following was helpful for me to deal with the message: _____________________

____________________________________________________________________

____________________________________________________________________

**MAIN STUDY**

1. Please find below some ways to deal with such a message. Please mark how the statements apply to you.

|  | disagree strongly | disagree | agree | agree strongly |
| --- | --- | --- | --- | --- |
| I do not think about it |  |  |  |  |
| I often think about the recipient or the family of the recipient |  |  |  |  |
| I have thought about my own family more often since I know the recipient has died |  |  |  |  |
| I talk to relatives and friends |  |  |  |  |
| I want to contact DKMS |  |  |  |  |
| I want to talk to other donors |  |  |  |  |
| Nevertheless, I feel happy for having donated |  |  |  |  |

The following was helpful for me to deal with the message: _____________________

____________________________________________________________________

____________________________________________________________________

1. Imagine you would be asked again to donate stem cells for another patient. Would you do it?

O Yes

O No

O I do not know

- - - 1. **If not**, why? __________________________________________________________

____________________________________________________________________

____________________________________________________________________
